# Risk of rebleeding from gastroesophageal varices after initial treatment with cyanoacrylate; a systematic review and pooled analysis

**DOI:** 10.1186/s12876-020-01333-9

**Published:** 2020-06-09

**Authors:** Zixuan Hu, Decai Zhang, Joel Swai, Tao Liu, Shaojun Liu

**Affiliations:** 1 Department of Gastroenterology, The Third Xiangya Hospital of Central South University, Changsha city, Hunan P.R. China; 2 Department of Nephrology and Rheumatology, The Third Xiangya Hospital of Central South University, Changsha city, Hunan P.R. China; 3 Department of Internal Medicine, Benjamin Mkapa Hospital, Dodoma city, East-Africa Tanzania; 4 Department of Cardiothoracic Surgery, The Third Xiangya Hospital of Central South University, Changsha city, Hunan P.R. China

**Keywords:** Cyanoacrylate, Tissue adhesive, Endoscopic hemostasis, Esophageal varices, Gastric varices, Rebleeding

## Abstract

**Background:**

Cyanoacrylate alone or in combination with other interventions, can be used to achieve variable rates of success in preventing rebleeding. Our study aims to assess the pooled risk of gastric and esophageal varices rebleeding after an initial treatment with cyanoacrylate alone and/or in combination with other treatments, by a systematic review of the literature and pooled analysis.

**Methods:**

PubMed, EMBASE, SCOPUS, and the Cochrane library were searched for studies that reported the risk of rebleeding during the follow-up period after treatment of gastric or esophageal varices with either cyanoacrylate alone or in combination with other treatments. Standard error, upper and lower confidence intervals at 95% confidence interval for the risk were obtained using ***STATA Version 15*** which was also used to generate forest plots for pooled analysis. The random or fixed effect model was applied depending on the heterogeneity (I^2^).

**Results:**

A total of 39 studies were found to report treatment of either gastric or esophageal varices with either cyanoacrylate alone or in combination with other treatments. When gastric varices are treated with cyanoacrylate alone, the risk of rebleeding during the follow-up period is 0.15(Confidence Interval: 0.11–0.18). When combined with lipiodol; polidocanol or sclerotherapy the rebleeding risks are 0.13 (CI:0.03–0.22), 0.10(CI:0.02–0.19), and 0.10(CI:0.05–0.18), respectively. When combined with percutaneous transhepatic variceal embolization; percutaneous transhepatic variceal embolization; endoscopic ultrasound guided coils; or with ethanolamine, the rebleeding risk are 0.10(CI:0.03–0.17), 0.10(CI:0.03–0.17), 0.07(CI:0.03–0.11) and 0.08(CI:0.02–0.14), respectively.

When esophageal varices are treated with cyanoacrylate alone, the risk of rebleeding is 0.29(CI:0.11–0.47). When combined with percutaneous transhepatic variceal embolization; sclerotherapy; or band ligation, the risks of rebleeding are 0.16(CI:0.10–0.22), 0.12(CI:0.04–0.20) and 0.10(CI:0.04–0.24), respectively. When combined with a transjugular intrahepatic portosystemic shunt; or ethanolamine, the risks of rebleeding are 0.06(CI: − 0.01-0.12) and 0.02 (CI: − 0.02-0.05), respectively.

**Conclusion:**

In treating both gastric and esophageal varices, cyanoacrylate produces better results in terms of lower risk of rebleeding when combined with other treatments than when used alone. The combination of cyanoacrylate with ethanolamine or with endoscopic ultrasound guided coils produces the lowest risk of rebleeding in esophageal and gastric varices, respectively. We call upon randomized trials to test these hypotheses.

## Background

Liver cirrhosis is the leading cause of portal hypertension which in turn, leads to portal hypertension and gastrointestinal varices. Up to 17% of liver cirrhosis patients will develop esophageal varices, while 15% will develop gastric varices. Up to 30%, gastroesophageal varices will bleed within 2 years [1]. Bleeding from varices is one among gastrointestinal emergencies that account for the majority of mortalities and morbidities among portal hypertension patients despite the cause [2]. About 50 to 80% of patients who survive the first episode of variceal hemorrhage will have a recurrent early or late rebleeding episode [3]. Up to 20% of patients with a rebleeding episode will not survive [4].

From older literature, half of the variceal hemorrhages would stop spontaneously however, the risk of rebleeding and mortality increases significantly [5]. Current studies, however, report that in patients with cirrhosis Child-Pugh of class C or with hepatic venous pressure of higher than 20 mm Hg are less likely to spontaneous stoppage of bleeding. These patients would require interventional hemostatic measures with pharmacological drugs such as octreotide, somatostatin and beta blockers; endoscopic sclerotherapy, band ligation, or tissue adhesives injection; and/or shunting by surgery or by transjugular intrahepatic portosystemic shunt to achieve hemostasis. A selective combination of these approaches has also been reported [1]. Different hemostatic approaches differ in terms of their success rates in achieving hemostasis, preventing rebleeding, and reducing mortality and morbidity. With advancing technology, each approach has evolved, and tissue adhesives have increasingly being used as the first line of therapy during the last decades [6, 7].

Also known as “tissue glue”, tissue adhesives were approved by the United States of America’s Food and Drug Authority in 1998, however, there have been previous studies reporting their use as back as the year 1981 [8]. Primarily containing n-butyl-2 cyanoacrylate or 2-octyl cyanoacrylate, tissue adhesives are liquid monomers that undergo chemical reactions upon contact with moisture, to form polymers that can strongly attach to tissue [9]. Despite a number of reported complications associated with their use such as embolism and needle impaction [10], cyanoacrylate has been reported to have higher hemostasis and lower rebleeding rates than traditional band ligation and sclerotherapy in gastroesophageal varices [2]. Moreover, they have been reported to have antibiotic activity towards gram-positive bacteria [11].

Cyanoacrylate can be used alone or in combination with other interventions, to achieve variable rates of successes in hemostasis, reducing mortality and prevention of rebleeding. Our study was aimed at assessing the overall risk of gastroesophageal rebleeding after an initial treatment with cyanoacrylate alone and/or in combination with other treatments, by a systematic review of literature and pooled analysis.

## Methods

### Eligibility criteria

The current study involved participants with bleeding gastroesophageal varices who underwent hemostasis by cyanoacrylate injection alone or in combination with other treatments. Observational and interventional studies reporting the risk of rebleeding after hemostasis treatment were included. Extending the external validity, eligible English published literature from across the world were included.

### Information sources

Four online databases, namely PubMed, EMBASE, SCOPUS, and the Cochrane library were systematically searched with no time range specified. Secondary referencing of eligible studies extended the search scope. The last search was conducted on 4th March 2020.

### The search

Advanced search tools employing MeSH and keywords, were utilized in all three online databases. Using PubMed, advanced search was done as; (cyanoacrylate [MeSH Terms]) AND endoscopic hemostasis [MeSH Terms]) AND esophageal varices [MeSH Terms]) OR gastric varices [MeSH Terms]) AND reble*. The search was repeated as; (adhes*) AND endosc*) AND varic*) AND reble*. The searches were independently performed by two authors; ZH and JS. Results were exported to *EndNote X9 (Builld 12,062)* which kept track of references.

### Study selection process

**Fig. 1:**
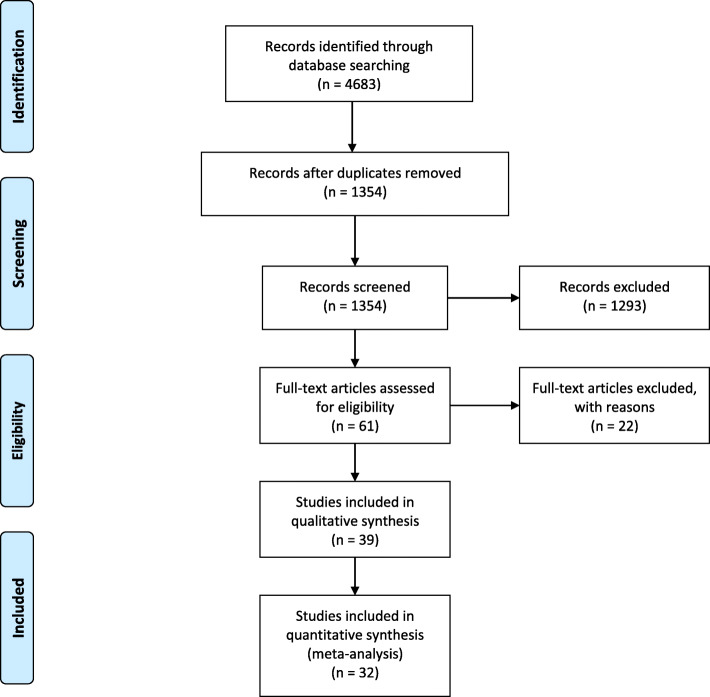
PRISMA 2009 flow diagram

Two authors screened titles and abstracts of all articles from online database searches to identify the most relevant articles in line with our study question. The relevant articles were sought for full texts and finally included studies were identified after thorough reading full text articles to assess inclusion and exclusion criteria. This process was done by two authors; ZH and JS with the third author, TL assisting to resolve discrepancies. The search, screening, and study identification process are summarized in Fig. 1.

### Data extraction

Before the data extraction process from full-text articles meeting eligibility criteria for inclusion, assessment for methodological biases was done by using the Joanna Briggs institute meta-analysis of statistics assessment and review instrument. PRISMA [12](preferred reporting items for systematic reviews and meta-analyses) tool was used to minimize reporting bias upon the write-up of this study.

Data collected included author name, year of publication, country of study, study design, comparison groups involved, varicose lesion location, study sample size, definitive diagnoses, number/ proportion of rebleeding events among followed up patients, the name of tissue adhesive utilized, treatment urgency, extent of live damage prior to treatment and follow-up duration. This was independently performed by two authors, namely; ZH and DZ with SL to resolve discrepancies. The current study had one outcome, the risk of rebleeding.

### Analysis

The risk of rebleeding among gastric and esophageal varices patients were analyzed separately. Moreover, the risk of rebleeding in gastric or esophageal varices groups was analyzed separately depending on whether the cyanoacrylate was utilized alone or in combination with other treatments. This gave rise to five separate analyses on which quantitative analysis was conducted: [1] Analyzing the pooled risk of rebleeding in gastric varices treated with cyanoacrylate alone [2]; analyzing the pooled risk of rebleeding in esophageal varices treated with cyanoacrylate alone [3]; analyzing the pooled risk of rebleeding in gastric varices treated with cyanoacrylate with ethanolamine [4]; analyzing the pooled risk of rebleeding in gastric varices treated with cyanoacrylate with endoscopic ultrasound guided coils; and [5] analyzing a pooled risk of rebleeding in esophageal varices treated with cyanoacrylate with percutaneous transhepatic variceal embolization. A qualitative narrative (i.e. descriptive) approach was utilized in assessing the risk of rebleeding in gastroesophageal varices treated with cyanoacrylate with sclerotherapy as the eligible studies involved different participants.

The risk of rebleeding was calculated dividing the number of patients rebleeding during the follow-up period after endoscopic hemostasis by the total number of patients that initially underwent the endoscopic hemostasis procedure. The denominator did not include patients lost during the follow up. Standard error, upper and lower confidence intervals (at 95% confidence interval) for the risk, were obtained from the “generate command” in computer software ***STATA Version 15*** which was also used to generate forest plots for pooled analysis. The software was customized to a random or fixed effect model depending on the heterogeneity (I^2^) of the studies when analyzing the outcomes. The fixed effect model was used when I^2^ was less than 50% and the random effect model was used when I^2^ was more than 50% indicating significant heterogeneity.

### Assumptions

Participants were considered to have been correctly diagnosed with upper gastrointestinal bleeding due to gastric or esophageal varices, and not due to other causes such as Mallory-Weiss tear or gastritis. Despite the country under which treatment was given, all patients were considered to have received standard care.

## Results

A total of sixty (60) studies that seemed to be relevant to our study basing on screening titles and abstract, were sought for full texts. Five of these were eliminated after thorough full-text reading. *Webb* et al. (1981) [8] did not report our outcome of interest; *Datta* et al. (2003) [13] and *Smith* et al. (2014) [14] utilized fibrin glue; *Noh* et al. (2004) [15] and *Zhang* et al. (2007) [16] used Korean and Chinese language, respectively. A total of 39 studies were included in the systematic review while 32 studies were pooled for statistical analysis.

### Characteristics of included studies

Table 1 illustrates the characteristics of all included studies in our pooled analysis. These were published between the years 1989 and 2019 from countries in Africa, Europe, Asia, and North America. Eleven studies were retrospective observational; 16 were prospective observational; two were case series, and ten were randomized clinical trials. Thirteen studies were comparative, one arm of which was cyanoacrylate. Eleven studies were non-comparative involving only cyanoacrylate outcome assessment while of the two studies, one involved comparing different doses of cyanoacrylate (i.e. 0.5mls versus 1.0mls) while another compared diluted versus undiluted cyanoacrylate. Follow-up duration after treatment with cyanoacrylate ranged from 6 weeks to 15 years in another study. One study did not report the duration of follow-up.

**Table 1:** Study characteristics

Author (Year)	Country of study	Study design	Comparison groups	Lesion; precise location	Diagnoses	Liver function/ extent of cirrhosis before treatment - number of patients	Sample size; number of participants rebled (%)	Treatment urgency	Follow-up duration
Ramond (1989) [17]	France	Case series	Butyl cyanoacrylate versus Sclerosant	Gastric; Unspecified	Cirrhosis; Portal vein thrombosis	Child-Pugh: A-12; B-11; C-4	27; 10 (37%)	Emergency and elective	1–38 Months (Mean: 14.7 ± 11.0)
Oho 1995 [18]	Japan	Randomized trial	Ethanolamine oleate or butyl cyanoacrylate	Gastric; cardiac and fundal.	Gastric varices	Child-Pugh: A-0; B-17; C-12	29; 9 (31%)	Emergency	14 months
D’Imperio 1996 [19]	Italy	Prospective	N-butyl-2- cyanoacrylate	Esophageal; Gastric; fundal Duodenal;	Upper gastrointestinal tract varices	Child-Pugh: A-17; B-37; C-23	24; 2 (3.7%) from gastric varices; 54; 0 (0%) from duodenal varices; Esophageal not reported	Emergency and elective	6 Months
Omar 1998 [20]	Egypt	Prospective trial	Polidocanol, ethanolamine, cyanoacrylate	Esophageal;	Schistosoma hepatic fibrosis	Data not accessed	60; not reported	Emergency	Not accessed
Kind 2000 [21]	Italy	Retrospective	One arm study: Bucrylate	Gastric; cardia and fundus	Gastric varices	Child-Pugh: A-8; B-64; C-101	174; 27 (15.52%)	Emergency	12 years
Evrad 2003 [22]	Belgium	Retrospective	N-butyl-2- cyanoacrylate versus Propranolol	Esophageal; unspecified Gastric;	Esophagogastric varices	Unspecified	16; 4 (25%); Esophageal 5; 2 (40%) Gastric	Emergency	6 weeks
Noophun 2005 [23]	Thailand	Prospective	One arm study: cyanoacrylate	Gastric; fundus	Gastric varices	Child-Pugh: A-6; B-11; C-7	24; 10 (41.67%)	Emergency and elective	Minimum of 4 weeks
Tan 2006 [24]	Taiwan	Prospective	Band ligation Versus N-butyl-2- cyanoacrylate	Gastric; fundus, antrum and isolated	Liver cirrhosis	Child-Pugh: A-13; B-26; C-10	49; 11 (22.45%) from cyanoacrylate group	Emergency	680.67 ± 710.54 days
Cheng 2007 [25]	China	Retrospective	One arm study: N-butyl-2- cyanoacrylate	Gastric; cardia and fundus	Gastric varices	Child-Pugh: A-194; B-254; C-134	635; 44 (8%)	Emergency	Up to 10 years
Kuo 2007 [26]	China	Randomized trial	Histoacryl versus Histoacryl + hypertonic glucose solution	Gastric;	Gastric varices	Child-Pugh: A-20; B-36; C-11	67; 2 (5.9%) from histoacryl-alone group	Emergent and elective	37.9 ± 18.5 months
Hong 2009 [27]	Korea	Randomized trial	Endoscopic N-butyl-2-cyanoacrylate injection versus balloon-occluded retrograde transvenous obliteration	Gastric; unspecified	Gastric variceal hemorrhage	Child-Pugh: A-3; B-8; C-3	27; 10 (71.43%) from the N-butyl-2-cyanoacrylate group	Emergent and elective	Up to 17 Months
Hou 2009 [28]	Taiwan	Randomized trial	0.5 mL Versus 1.0 mL of cyanoacrylate	Gastric; cardiac, fundal and undetermined	Gastric variceal hemorrhage	Mean ± standard deviation of Child-Pugh scores: 0.5 mL group 7.61 ± 1.82 1.0 mL group 7.79 ± 2.27	46; 14 (29.79%) from the 0.5mls group; 44; 17 (38.64%) from the 1 ml group	Emergent and elective	Up to 2 years
Procaccini 2009 [29]	USA	Retrospective	Cyanoacrylate versus TIPS	Gastric; unspecified	Gastric variceal hemorrhage	Model for End-Stage Liver Disease (MELD), Mean ± standard deviation score: Cyanoacrylate, 15.4 ± 6.3; TIPS, 15.7 ± 8.3	105; 13 (21.31%) from the Cyanoacrylate group	Emergent and elective	Up to 1 year
Rivet 2009 [30]	France	Prospective	Cyanoacrylate versus Band ligation	Esophageal;	Portal hypertension due to portal vein thrombosis, biliary atresia and antitrypsin deficiency	Pediatric End-stage Liver Disease (PELD) model, mean ± 20.1 ± 9.9	8; 3 (37.5%) from cyanoacrylate group	Emergent and elective	10.6 weeks
Cheng 2010 [31]	China	Retrospective	Butyl cyanoacrylate	Gastric; cardiac and fundus	Gastric varices due to viral hepatitis and others	Child-Pugh: A-244; B-297; C-179; unspecified-33	753; 33 (4.38%)	Emergent and elective	Up to 6 months after initial endoscopy
Choudhuri 2010 [32]	India	Prospective	N-butyl-2-cyanoacrylate	Gastric; unspecified	Gastric variceal hemorrhage	Child-Pugh: A-40; B-62; C-40	170; 23 (14.56%)	Emergent and elective	30.7 + 17.2 months
Mishra 2010 [33]	India	Prospective	Cyanoacrylate versus beta blocker	Gastric; cardiac, fundus and isolated Esophageal;	Gastric varices	Child-Pugh: A-4; B-12; C-17	33; 3 (9.09%) from the cyanoacrylate group	Emergency	26 Months
Soga 2010 [34]	Japan	Case report	N-butyl-2-cyanoacrylate	Gastric; Duodenal;	Gastroduodenal varices	Unspecified	1; 0 (0%)	Elective	53 days
Binmoellar 2011 [35]	USA	Retrospective	N-butyl-2-cyanoacrylate	Gastric; fundus	Gastric varices; Non variceal lesion	MELD score < 10 mean ± standard deviation, 11 ± 40.7; MELD score 11–18 mean ± standard deviation, 12 ± 44.4; MELD score 19–24, mean ± standard deviation, 3 ± 11.1	24; 0 (0%) from gastric variceal group	Emergent and elective	193 (24–589) days
Kang 2011 [36]	Korea	Retrospective	N-butyl-2-cyanoacrylate cyanoacrylate	Gastric; cardiac, fundal and isolated	Gastric varices	Child-Pugh: A-42; B-59; C-26	127; 29 (22.83%)	Emergent and elective	1 year
Liao 2013 [37]	Taiwan	Prospective	Cyanoacrylate	Gastric; unspecified	Gastric varices	Child-Pugh: A-16; B-13; C-6	69; 10 (14.49%)	Emergency and elective	More than 30 months
Tantau 2013 [38]	Romania	Prospective	Cyanoacrylate versus Band ligation	Gastric; cardiac and fundus	Gastric varices	Child-Pugh: A-11; B-18; C-8	37; 6 (31.58%) from the Cyanoacrylate group	Emergency and elective	27.26 ± 214.16 days
Al-Bawardy 2016 [39]	USA	Retrospective	2-octyl cyanoacrylate	Gastric; fundal	Gastric Variceal Hemorrhage	MELD score median value, 11	95; 8 (8.42%)	Emergency	Up to 15 years
Singh 2016 [10]	India	Prospective	Diluted versus undiluted cyanoacrylate	Gastric; cardiac, fundus and isolated	Gastric Variceal Hemorrhage	Child-Pugh: A-9; B-15; C-6	30; 5 (16.67%)	Emergency and elective	Up to one year
Liu 2019 [40]	China	Prospective	Cyanoacrylate with versus without antibiotic	Gastric; unspecified	Gastric varices	Child-Pugh: A-76; B-31; C-0	107; 106 (99.07%)	Emergency	4.59 ± 1.63; 4.30 ± 1.48 Days
Xiaoqing 2019 [2]	China	Prospective	Cyanoacrylate versus cyanoacrylate + lauromacrogol	Gastric; cardiac, fundus and isolated	Gastric varices	Child-Pugh: A-27; B-74; C-9	130; 8 (12.90%) from the Cyanoacrylate group	Emergency	38.8 months for Cyanoacrylate group
Thakeb 1995 [41]	Egypt	Randomized trial	N-butyl-2-cyanoacrylate plus ethanolamine oleate 5% versus ethanolamine alone	Gastric; Unspecified Esophageal;	Gastroesophageal varices	Child-Pugh: A-16; B-33; C-9	57; 3 (5.26%) from the gastric varices; 59; 1 (1.69%) from the esophageal varices group.	Emergency and elective	Up to 32 months
Maruyama 2010 [42]	Japan	Retrospective	Cyanoacrylate plus ethanolamine	Gastric; fundus	Gastric varices	Child-Pugh: A-2; B-4; C-4	20; 10 (50%)	Emergency	28.1 months
Bhat 2016 [43]	United States of America	Retrospective	Cyanoacrylate and coils guided by endoscopic ultrasound	Gastric; fundal	Gastric varices	MELD score < 10, 31; MELD score 11–18, 70; MELD score 19–24, 10; MELD score > 24, 3	125; 10 (8%)	Elective	Median: 436 days;
Robles-Medranda 2019 [44]	Ecuador	Prospective	Cyanoacrylate and coils guided by endoscopic ultrasound	Gastric; cardiac, fundus and isolated	Gastric varices	Child-Pugh: A-28; B-2; C-17	30: 1 (3.7%)	Emergency and elective	Up to 12 months
Zhang 2007 [16]	China	Randomized trial	Cyanoacrylate with percutaneous transhepatic variceal embolization	Esophageal;	Esophageal varices	Data not accessed	92; 14 (16%)	Emergency and elective	Mean: 31.5 months
Zhang 2008 [45]	China	Randomized trial	Cyanoacrylate with percutaneous transhepatic variceal embolization	Esophageal; cardiac, fundus and isolated	Esophageal varices	Child-Pugh: A-10; B-25; C-17	52; 8 (15.38%)	Emergency and elective	Median: 25 months
Tian 2011 [46]	China	Prospective	Cyanoacrylate with percutaneous transhepatic variceal embolization	Gastric; cardiac, fundus, isolated	Gastric varices	Child-Pugh: A-24; B-31; C-17	71; 7 (9.86%)	Emergency and elective	Mean; 24.2 ± 12.4 months
Feritis 1995 [47]	Greece	Randomized trial	Cyanoacrylate with sclerotherapy	Esophageal;	Esophageal varices	Child-Pugh: A-12; B-83; C-35	126; 8 (11.94%)	Emergency	30 days
Dhiman 2002 [48]	India	Prospective	Cyanoacrylate with sclerotherapy	Gastric; fundal	Gastric varices	Child-Pugh: A-5; B-5; C-3	29; 3 (10.34%)	Emergency and elective	Up to 6 months
Shi 2014 [49]	China	Retrospective	Transjugular intrahepatic portosystemic shunt alone versus combined with cyanoacrylate	Esophageal;	Esophageal Variceal Bleeding	Child-Pugh: A-27; B-57; C-17	53; 3 (5.66%)	Emergency	35.8 months
Ma 2018 [50]	China	Prospective	Combined cyanoacrylate with balloon-occluded retrograde transvenous obliteration	Gastroesophageal;	Gastroesophageal varices	Child-Pugh: A-16; B-10; C-2	28; 8 (31%)	Elective	90 days
Dai 2017 [51]	China	Randomized trial	Band ligation alone versus in combination with cyanoacrylate	Gastroesophageal;	Gastroesophageal varices	Data not accessed	97; 7 (14.29%)	Emergency and elective	20 months
Zeng 2017 [52]	China	Randomized trial	Cyanoacrylate plus polidocanol versus cyanoacrylate plus lipiodol in	Gastric; cardiac, fundus and isolated	Gastric varices	Child-Pugh: A-50; B-44; C-4	96; 11 (11.70%)	Emergency and elective	6 months

A total of 39 studies reported 3630 who had either gastric or esophageal variceal and underwent hemostasis with cyanoacrylate alone or in combination with other treatments. A total of 497 had gastric or esophageal recurrent bleeding episodes during the follow-up period.

### Pooled risk of rebleeding in gastric varices treated with cyanoacrylate alone

**Fig. 2:**
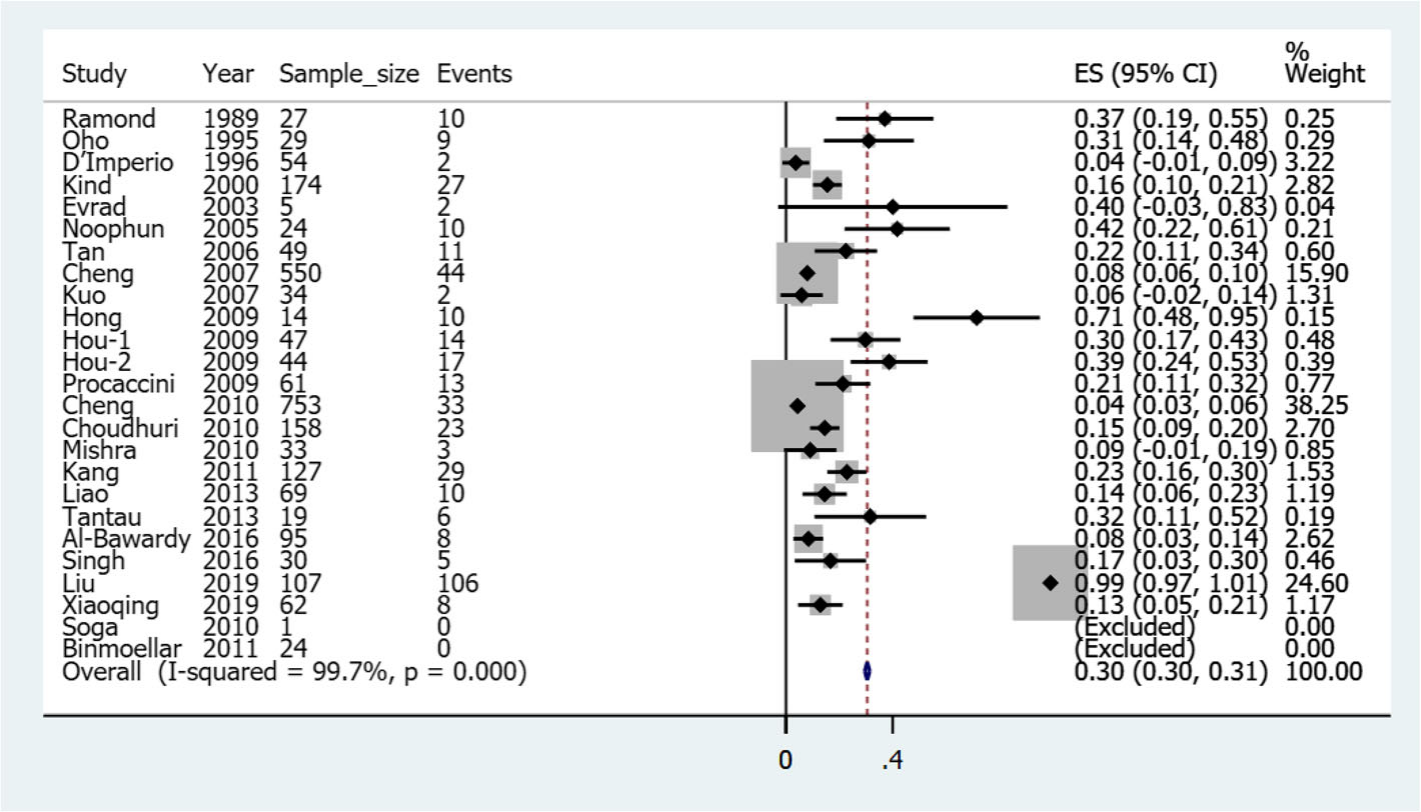
A forest plot of the pooled risk of rebleeding for gastric varices after cyanoacrylate treatment

Figure 2 illustrates a forest plot of the pooled risk of rebleeding for gastric varices after cyanoacrylate treatment. A total of 25 studies reported 2590 gastric variceal patients, of whom 402 had had rebleeding after initial treatment with cyanoacrylate hemostasis. The risk ranged from a minimum of 0.04 (4%) to a maximum of 0.99 (99%) in another study. Two studies were excluded for not having rebleeding incidences during the follow up period. The pooled overall risk of rebleeding was 0.30 (confidence interval: 0.30–0.31).

**Fig. 3:**
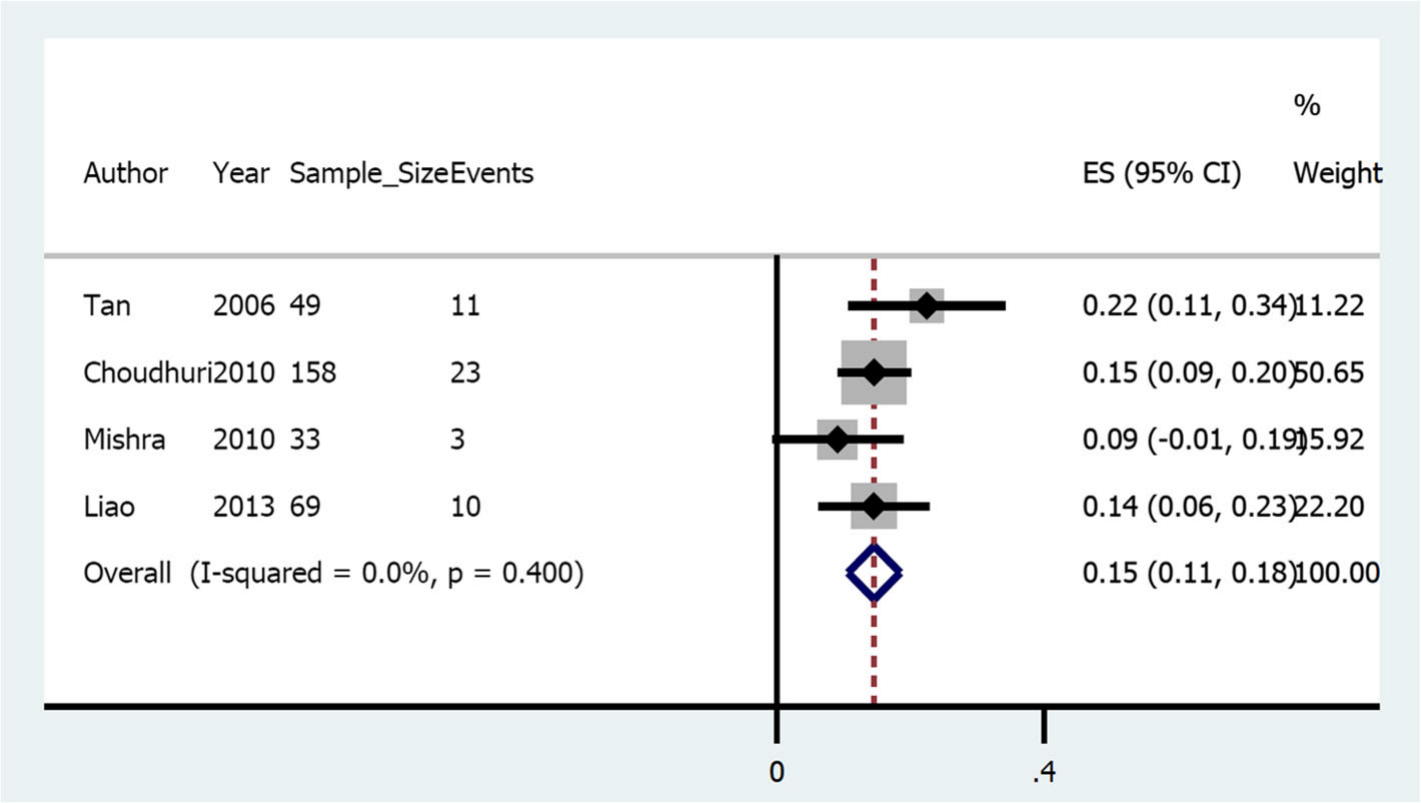
Sensitivity analysis-forest plot of the pooled risk of rebleeding for gastric varices after the elimination of peculiar studies

There was a significant heterogeneity observed with I^2^ of 99.7%, *p*-Value< 0.05. This led us to conduct sensitivity analysis, eliminating peculiar studies from the analysis. Figure 3 illustrates a sensitivity analysis forest plot of the pooled risk of rebleeding for gastric varices after the elimination of peculiar studies. *Ramond* et al. (1989) [17] and *Soga* et al. (2010) [34] were case series and case report respectively; *D’Imperio* et al. (1996) [19], *Omar* et al. (1998) [20], *Noophun* et al. (2005) [23], *Rivet* et al. (2009) [30], *Cheng* et al. (2010) [31], *Binmoellar* et al. (2011) [35] and *Tantau* et al. (2013) [38] had less than one-year of follow-up; while *Kind* et al. (2000) [21], *Tan* et al. (2006) [24], *Procaccini* et al. (2009) [29], *Choudhuri* et al. (2010) [32], *Mishra* et al. (2010) [33], *Liao* et al. (2013) [37], *Singh* et al. (2016) [10], *Cheng* et al. (2007) [25], *Kuo* et al. (2007) [26], *Huo* et al. (2009) [28], *Kang* et al. (2011) [36], *Al-Baward* et al. (2016) [39] and *Xiaoqing* et al. (2019) [2] were excluded by meta-regression. *Evrad* et al. (2003) [22], *Hong* et al. (2009) [27], *Soga* et al. (2010) [34] and *Liu* et al. (2019) [40] were excluded because their findings did not fulfill normality test criteria for calculation of confidence interval (i.e.N(1-Pe) ≥10). Moreover, regarding follow-up time, *Sigh* et al. (2016), *Procaccini* et al. (2009), and Kind et al. (2000) were excluded for distinct follow-up times. Each of the 4 remaining studies had an estimate of 2 years of follow-up. The resulting overall pooled risk was 0.15 (Confidence interval: 0.11–0.18) with no significant heterogeneity (i.e. I^2^ = 0.0%, *p*-Value = 0.4).

### Pooled risk of rebleeding in esophageal varices treated with cyanoacrylate alone

**Fig. 4:**
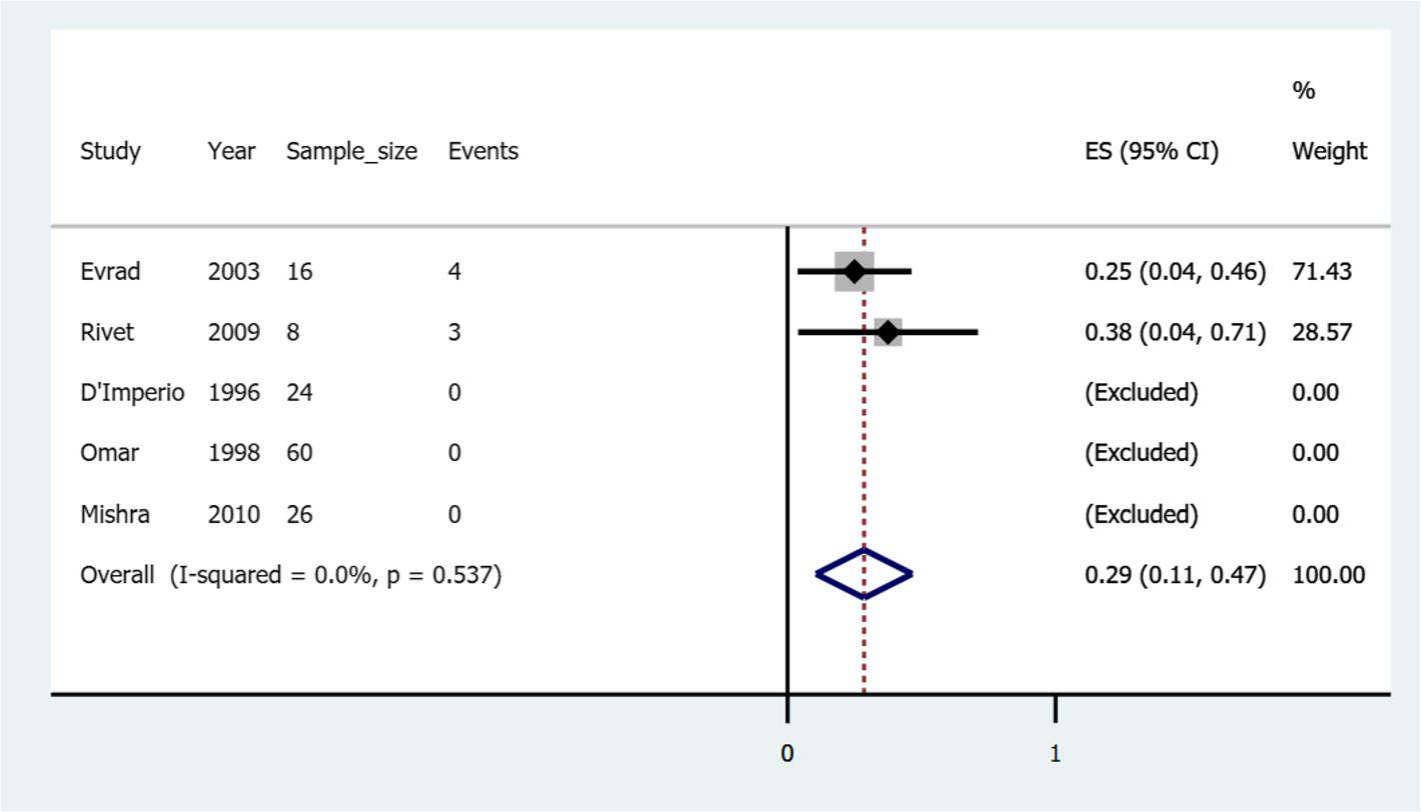
A forest plot of the pooled risk of rebleeding of esophageal varices after cyanoacrylate treatment

Figure 4 illustrates a forest plot of the pooled risk of rebleeding for esophageal varices after cyanoacrylate treatment. A total of five studies reported 134 esophageal variceal patients, 7 of whom had had rebleeding after initial treatment with cyanoacrylate hemostasis. The risk of rebleeding ranged from a minimum of 0.25 (25%) to a maximum of 0.38 (99%) in another study. Three studies were excluded for not having rebleeding incidences during the follow up period. The pooled overall risk of rebleeding was 0.29 (confidence interval: 0.11–0.47). There was no significant heterogeneity observed; I^2^ of 0.0%, *p*-Value = 0.53.7).

### Pooled risk of rebleeding in gastric varices treated with cyanoacrylate with ethanolamine

**Fig. 5:**
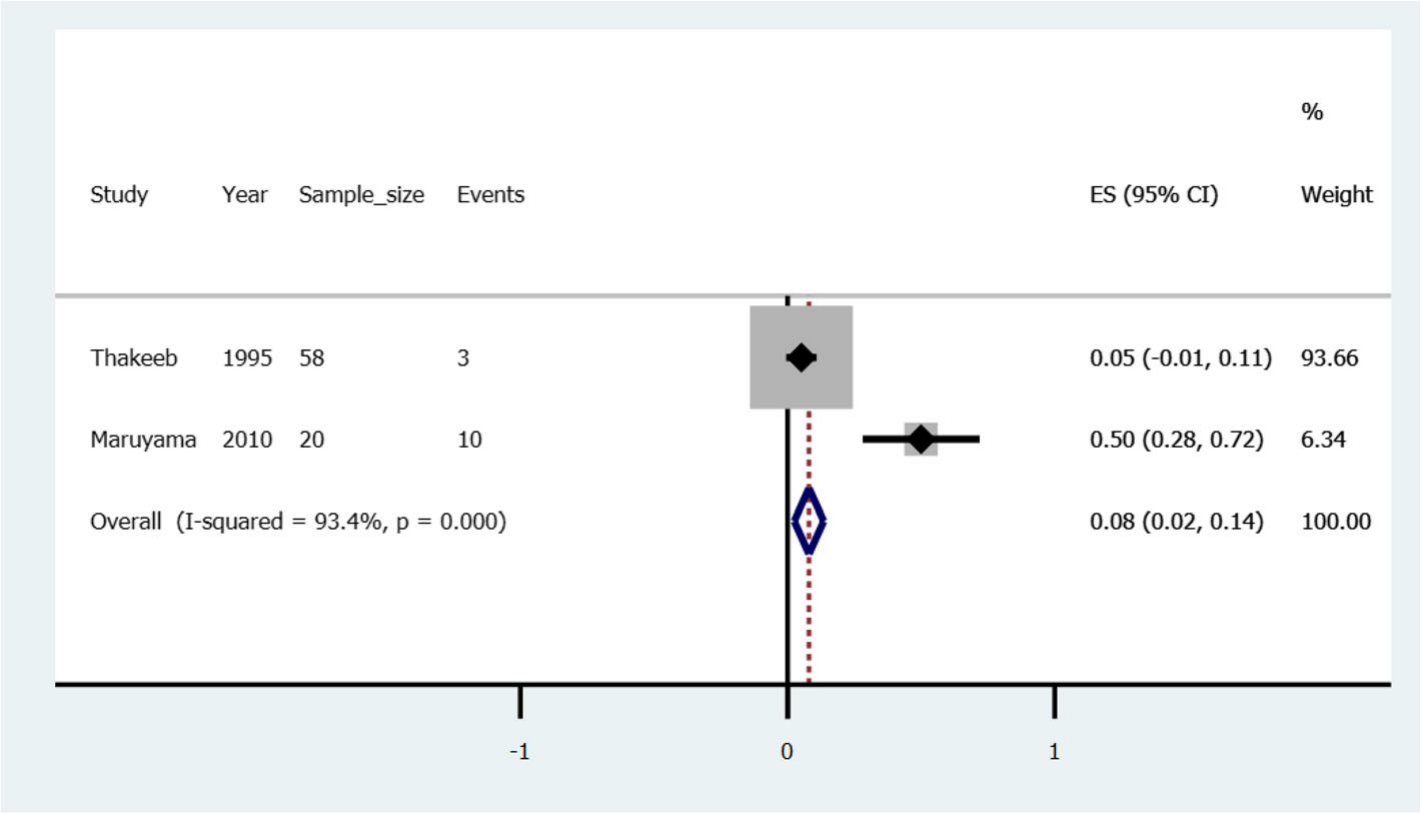
A forest plot of the pooled risk of rebleeding in gastric varices treated with a combination of cyanoacrylate with ethanolamine

Two studies illustrated treatment with a combination of cyanoacrylate and ethanolamine; *Thakeeb* et al. (1995) [41] and *Maruyama* et al. (2010) [42]. *Thakeeb* reported 3 (i.e. risk = 0.052) rebleeding events among gastric variceal patients; and one (risk = 0.017) rebleeding events among esophageal varices patients. *Maruayama* reported 10 (i.e. risk =0.5) rebleeding events among gastric varices patients. Figure 5 illustrates a forest plot of pooled risk, 0.08(0.02–0.14) of rebleeding in gastric varices treated with a combination of cyanoacrylate with ethanolamine.

### Pooled risk of rebleeding in gastric varices treated with cyanoacrylate with endoscopic ultrasound guided coils

**Fig. 6:**
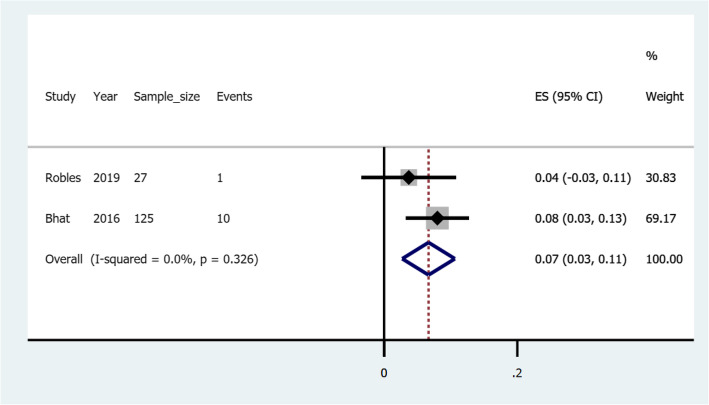
A forest plot of the pooled risk of rebleeding in gastric varices treated with cyanoacrylate with endoscopic ultrasound guided coils

Two studies illustrated treatment with a combination of cyanoacrylate and coils guided by endoscopic ultrasound; *Bhat* et al. (2016) [43] and *Robles-Medranda* et al. (2019) [44]. *Bhat* et al. (2016) reported 10 rebleeding events out of 125 gastric varices patients who were followed-up. This corresponds to the risk of 0.08 (Confidence interval: 0.03–0.13). *Robles-Medranda* et al. (2019) reported 1 rebleeding event out of 27 gastric varices patients, which corresponds to the risk of 0.04 (Confidence interval: − 0.03-0.11). Figure 6 illustrates a forest plot of the pooled risk of rebleeding in gastric varices treated with cyanoacrylate with endoscopic ultrasound guided coils.

### Pooled risk of rebleeding in esophageal varices treated with cyanoacrylate with percutaneous transhepatic variceal embolization

**Fig. 7:**
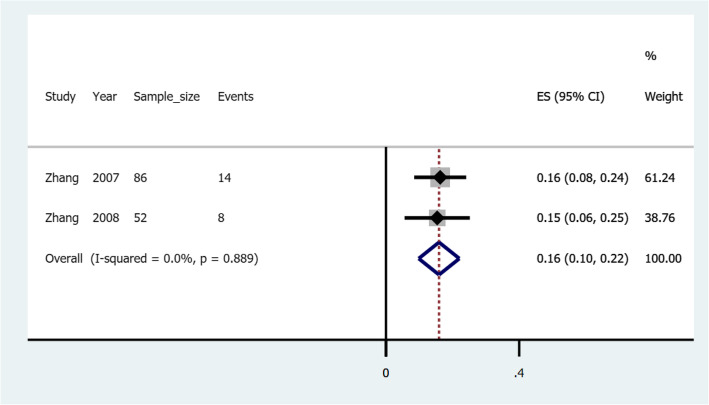
A forest plot of the pooled risk of rebleeding in esophageal varices treated with cyanoacrylate with percutaneous transhepatic variceal embolization

Three studies illustrated treatment with a combination of cyanoacrylate and percutaneous transhepatic variceal embolization in gastroesophageal varices; *Zhang* et al. (2007) [16] and *Zhang* et al. (2008) [45] involved esophageal varices patients and reported rebleeding risks of 0.16 (confidence interval: 0.08–0.24) and 0.15 (confidence interval: 0.06–0.25), respectively. *Tian* et al. (2011) [46] involved gastric varices patients and reported a rebleeding risk of 0.10(confidence interval: 0.03–0.17). Figure 7 illustrates a forest plot of the pooled risk of rebleeding in esophageal varices treated with cyanoacrylate with percutaneous transhepatic variceal embolization.

### Risk of rebleeding in gastroesophageal varices treated with cyanoacrylate with sclerotherapy

Two studies assessed the efficacy of a combination of cyanoacrylate and sclerotherapy in the treatment of gastroesophageal varices. In one study, *Feretis* et al. (1995) [47] compared the combination versus sclerotherapy alone in the treatment of esophageal varices and reported the risk for rebleeding in the combination group to be 0.12 (Confidence interval: 0.04–0.20). In another one arm study, *Dhiman* et al.(2002) [48] assessed the outcome of the combination therapy in the treatment of gastric varices and reported a risk of 0.10 (Confidence interval: 0.05–0.18). Forest plot was not constructed as the two studies involved different participants (i.e. gastric and esophageal varices).

### Other combination treatments with cyanoacrylate

In their study, *Shi* et al. (2014) [49] compared between transjugular intrahepatic portosystemic shunt alone versus combined with Cyanoacrylate for esophageal variceal bleeding. The combination therapy reduced the rebleeding risk to a third of one observed in transjugular intrahepatic portosystemic shunt alone. That is from 0.19 to 0.06, *p*-Value of 0.04. In another study, *Ma* et al. (2018) [50] combined cyanoacrylate with balloon-occluded retrograde transvenous obliteration in 28 patients with gastroesophageal varices and reported a rebleeding risk of 0.31 (confidence interval: 0.13–0.49).

*Dai* et al. (2017) [51] compared band ligation alone versus in combination with cyanoacrylate in the treatment of gastroesophageal varices. The risk of rebleeding in the combination therapy was reduced to a quarter that recorded in band ligation alone. That is from 0.56 to 0.14, p-Value< 0.01. *Zeng* et al. (2017) [52] compared two combinations; cyanoacrylate plus polidocanol versus cyanoacrylate plus lipiodol in the treatment of gastric varices. The later showed the risk of rebleeding of 0.13 (Confidence Interval: 0.03–0.22) as compared to 0.10 (Confidence interval: 0.02–0.19) in the polidocanol combination.

Table 2 summarizes risks of rebleeding in gastric and esophageal varices when treated with cyanoacrylate alone or in combination with other treatments as discussed earlier.

**Table 2:** Risks of rebleeding in gastric and esophageal varices after treatment with cyanoacrylate alone or in combination with other treatments

Hemostasis treatment type	Pooled risk of gastric varices rebleeding (confidence interval)	Pooled risk of esophageal varices rebleeding (confidence interval)
Cyanoacrylate alone	0.15 (0.11–0.18)	0.29 (0.11–0.47)
Cyanoacrylate combined with ethanolamine	0.08 (0.02–0.14)	0.02 (− 0.02–0.05).
Cyanoacrylate combined with endoscopic ultrasound guided coils	0.07 (0.03–0.11)	–
Cyanoacrylate combined with percutaneous transhepatic variceal embolization	0.10 (0.03–0.17) ^a^	0.16 (0.10–0.22)
Cyanoacrylate combined with transjugular intrahepatic portosystemic shunt	–	0.06(−0.01–0.12) ^a^
Cyanoacrylate combined with sclerotherapy	0.10 (0.05–0.18) ^a^	0.12 (0.04–0.20) ^a^.
Cyanoacrylate combined with band ligation	–	0.10 (0.04–0.24) ^a^
Cyanoacrylate combined with polidocanol	0.10 (0.02–0.19) ^a^	–
Cyanoacrylate combined with lipiodol	0.13 (0.03–0.22) ^a^	–
Cyanoacrylate combined with balloon-occluded retrograde transvenous obliteration	0.31 (0.13–0.49) ^b^	

Note: The values in the table are independently calculated and the table does not mean statistical comparison between them

Key: ^a^ Calculated from a single study (Not pooled); ^b^ Gastric or esophageal varices not specified (Gastroesophageal)

## Discussion

Through decades-long progressive improvements in the treatment of gastroesophageal varices, cyanoacrylate has evolved to be one of the favored first lines of treatment. The current study was aimed at utilizing a systematic review of literature and pooled analysis to assess the overall risk of gastroesophageal rebleeding after an initial treatment with cyanoacrylate alone and/or in combination with other treatments.

Following the treatment of gastric varices with cyanoacrylate alone, 25 studies demonstrated different risks of rebleeding from the minimum of 0.04 to a maximum of 0.99 in another study, with the overall pooled risk of 0.30 (confidence interval: 0.30–0.31). However, after getting rid of peculiar studies that increased heterogeneity, the resulting overall pooled risk was 0.15 (Confidence interval: 0.11–0.18). This risk of rebleeding coincides with that previously reported by *Hou* et al. (2009) [28] but differed from the majority of other studies. Authors believe that the reason for the differences among studies to be technological advancement with time. This can be demonstrated the majority of studies from the year 2010 forward having a lower risk of rebleeding than studies before 2010. Different sample sizes and different study methodologies could also explain the differences.

Esophageal varices treated with cyanoacrylate alone showed the risk of rebleeding ranging from 0.25 to 0.38 in different studies with the pooled overall risk of 0.29 (confidence interval: 0.11–0.47). Following a fewer number of studies, a meta regression could not be conducted. However, authors believe that the reason for the differences between studies to be due to different methodological approaches between the studies as *Rivet* et al. (2009) [30] followed up their patients for twice the duration used by *Evrad* et al. (2003) [22]. The authors of this study hypothesize that gastric varices respond better to cyanoacrylate as compared to esophageal varices in terms of lower risk of rebleeding. We call upon randomized clinical trials comparing the risk of rebleeding between gastric varices and esophageal varices treated with cyanoacrylate alone.

When cyanoacrylate is combined with ethanolamine in the treatment of gastric varices the pooled risk of rebleeding after treatment is 0.08 (Confidence interval: 0.02–0.14). The result aligns with that reported by *Thakeb* et al. (1995) but differs from *Maruyama* who reported a higher risk of 0.5. The difference is accounted for by fewer sample size by *Maruyama*. On the other hand, when the combination is used to treat esophageal varices the risk of rebleeding is 0.017(confidence interval: − 0.02-0.05). From an otherwise weak basis, we hypothesis that esophageal varices in contrast to gastric varices, respond better to the combination of cyanoacrylate and ethanolamine, in terms of lower risk of rebleeding. We call upon clinical randomized clinical trials to test this hypothesis.

From our findings, when cyanoacrylate is combined with endoscopic ultrasound guided coils to treat gastric varices the pooled risk of rebleeding is 0.07(confidence interval: 0.03–0.11). This finding is more or less similar to that reported by *Bhat* et al. (2016) [43] but is higher than that reported by *Robles-Medranda* et al. (2019) [44]. The reason for the differences could be explained by different sample sizes among studies pooled. One study had nearly five times the sample size used by the other.

When esophageal varices are treated with a combination of cyanoacrylate and percutaneous transhepatic variceal embolization the pooled risk of rebleeding is 0.16(confidence interval: 0.10–0.22). This is coinciding with findings previously reported by *Zhang* et al. (2007) [16]. In another study by *Tian* et al. (2011) [46] when the combination is used to treat gastric varices, the risk of rebleeding is 0.10(confidence interval: 0.03–0.17). We hypothesize that esophageal varices in contrast to gastric varices, respond better to the combination of cyanoacrylate and percutaneous transhepatic variceal embolization in terms of lower risk of rebleeding. The authors call upon randomized clinical trials to test this hypothesis.

The risk of rebleeding in gastric varices treated with cyanoacrylate with sclerotherapy was lower by 0.02 from that of esophageal varices treated with the same combination. The difference could partly be due to more or less the same number of sample sizes among the two studies descriptively analyzed. In combination with other treatments such as transjugular intrahepatic portosystemic shunt and balloon-occluded retrograde transvenous obliteration, it is evident that cyanoacrylate improves the efficacy of the treatment of gastroesophageal varices in terms of lowering rebleeding risk.

### Study limitations, measures taken, and recommendations

Our study search was limited to English published literature; involved pooling of studies with different sample sizes, different study designs, and different follow-up durations. As demonstrated by Child-Pugh or the model for end-stage liver disease (MELD) classifications, different studies involved participants with different extents of liver damage/cirrhosis. Despite a few studies involving either emergent [49] or elective [50] participants only, the majority of studies combined the two [16, 48]. Moreover, from older literature by Sarin et al. (1992) [53], the risk of rebleeding varied with lesion’s location on the gastric wall. From the study, isolated varices bled more often as compared to cardia and fundal varices.

These were thought to introduce heterogeneity in the pooled analysis. However, authors appraised eligible studies; performed sensitivity analyses, meta-regression, study exclusion, and used random effect models to deal with high heterogeneity among pooled studies. We also utilized PRISMA tools to minimize reporting biases.

We call upon robust randomized studies taking into account biases encountered in our study and adequately matching participants by the extent of liver damage/cirrhosis; treatment urgency whether elective or emergency; lesion location and follow-up duration.

## Conclusion

In treating both gastric and esophageal varices, cyanoacrylate produces better results in terms of lower risk of rebleeding when combined with other treatments than when used alone. The combination of cyanoacrylate with ethanolamine or with endoscopic ultrasound guided coils produces the lowest risk of rebleeding in esophageal and gastric varices, respectively. We call upon randomized trials to test these hypotheses.

## Data Availability

The datasets used and analyzed during the current study are available from the corresponding author on reasonable request.

## Abbreviations

PRISMA: Preferred reporting items for systematic reviews and meta-analyses
MeSH: Medical Subject Headings
ZH: Zixuan Hu (Author)
DZ: Decai Zhang (Author)
JS: Joel Swai (Author)
TL: Tao Liu (Author)
SL: Shaojun Liu (Author)

## References

[1] Habib A, Sanyal AJ (2007). Acute variceal hemorrhage. Gastrointest Endosc Clin N Am.

[2] Xiaoqing Z, Na L, Lili M, Jie C, Tiancheng L, Jian W (2019). Endoscopic cyanoacrylate injection with Lauromacrogol for gastric Varices: long-term outcomes and predictors in a retrospective cohort study. J Laparoendoscopic Advanced Surgical Techniques Part A.

[3] Burroughs AK, McCormick PA (1992). Prevention of variceal rebleeding. Gastroenterol Clin N Am.

[4] Mehmood T, Zia MQ, Latif A, Ansar S (2019). Mortality related factors in patients with Variceal bleeding with MELD score >/= 18. J College Physicians Surgeons--Pakistan.

[5] Prandi D, Rueff B, Roche-Sicot J, Sicot C, Maillard JN, Benhamou JP (1976). Life-threatening hemorrhage of the digestive tract in cirrhotic patients. An assessment of the postoperative mortality after emergency portacaval shunt. Am J Surg.

[6] de Franchis R (2010). Revising consensus in portal hypertension: report of the Baveno V consensus workshop on methodology of diagnosis and therapy in portal hypertension. J Hepatol.

[7] Garcia-Tsao G, Sanyal AJ, Grace ND, Carey W (2007). Prevention and management of gastroesophageal varices and variceal hemorrhage in cirrhosis. Hepatology (Baltimore, Md).

[8] Webb WA, McDaniel L, Johnson RC, Haynes CD (1981). Endoscopic evaluation of 125 cases of upper gastrointestinal bleeding. Ann Surg.

[9] Singer AJ, Quinn JV, Hollander JE (2008). The cyanoacrylate topical skin adhesives. Am J Emerg Med.

[10] Singh V, Singh R, Bhalla A, Sharma N (2016). Cyanoacrylate therapy for the treatment of gastric varices: a new method. J Dig Dis.

[11] Rushbrook JL, White G, Kidger L, Marsh P, Taggart TF (2014). The antibacterial effect of 2-octyl cyanoacrylate (Dermabond(R)) skin adhesive. J Infect Prev.

[12] Moher D, Liberati A, Tetzlaff J, Altman DG, The PG (2009). Preferred reporting items for systematic reviews and Meta-analyses: The PRISMA statement. PLoS Med.

[13] Datta D, Vlavianos P, Alisa A, Westaby D (2003). Use of fibrin glue (beriplast) in the management of bleeding gastric varices. Endoscopy..

[14] Smith MR, Tidswell R, Tripathi D (2014). Outcomes of endoscopic human thrombin injection in the management of gastric varices. Eur J Gastroenterol Hepatol.

[15] Noh DY, Park SY, Joo SY, Park CH, Lee WS, Joo YE (2004). Therapeutic effect of the endoscopic N-butyl-2-cyanoacrylate injection for acute esophagogastric variceal bleeding: comparison with transjugular intrahepatic portosystemic shunt. Korean J Gastroenterol.

[16] Zhang CQ, Liu FL, Xu HW, Feng K, Zhu Q, Zhang JY (2007). Percutaneous transhepatic varices embolization with cyanoacrylate in the treatment of varices. Zhonghua Yi Xue Za Zhi.

[17] Ramond MJ, Valla D, Mosnier JF, Degott C, Bernuau J, Rueff B (1989). Successful endoscopic obturation of gastric varices with butyl cyanoacrylate. Hepatology (Baltimore, Md).

[18] Oho K, Iwao T, Sumino M, Toyonaga A, Tanikawa K (1995). Ethanolamine oleate versus butyl cyanoacrylate for bleeding gastric varices: a nonrandomized study. Endoscopy..

[19] D'Imperio N, Piemontese A, Baroncini D, Billi P, Borioni D, Dal Monte PP (1996). Evaluation of undiluted N-butyl-2-cyanoacrylate in the endoscopic treatment of upper gastrointestinal tract varices. Endoscopy..

[20] Omar MM, Fakhry SM, Mostafa I (1998). Immediate endoscopic injection therapy of bleeding oesophageal varices: a prospective comparative evaluation of injecting materials in Egyptian patients with portal hypertension. J Egypt Soc Parasitol.

[21] Kind R, Guglielmi A, Rodella L, Lombardo F, Catalano F, Ruzzenente A (2000). Bucrylate treatment of bleeding gastric varices: 12 years' experience. Endoscopy..

[22] Evrard S, Dumonceau JM, Delhaye M, Golstein P, Deviere J, Le Moine O (2003). Endoscopic histoacryl obliteration vs. propranolol in the prevention of esophagogastric variceal rebleeding: a randomized trial. Endoscopy..

[23] Noophun P, Kongkam P, Gonlachanvit S, Rerknimitr R (2005). Bleeding gastric varices: results of endoscopic injection with cyanoacrylate at King Chulalongkorn Memorial Hospital. World J Gastroenterol.

[24] Tan PC, Hou MC, Lin HC, Liu TT, Lee FY, Chang FY (2006). A randomized trial of endoscopic treatment of acute gastric variceal hemorrhage: N-butyl-2-cyanoacrylate injection versus band ligation. Hepatology (Baltimore, Md).

[25] Cheng LF, Wang ZQ, Li CZ, Cai FC, Huang QY, Linghu EQ (2007). Treatment of gastric varices by endoscopic sclerotherapy using butyl cyanoacrylate: 10 years' experience of 635 cases. Chin Med J.

[26] Kuo MJ, Yeh HZ, Chen GH, Poon SK, Yang SS, Lien HC (2007). Improvement of tissue-adhesive obliteration of bleeding gastric varices using adjuvant hypertonic glucose injection: a prospective randomized trial. Endoscopy..

[27] Hong CH, Kim HJ, Park JH, Park DI, Cho YK, Sohn CI (2009). Treatment of patients with gastric variceal hemorrhage: endoscopic N-butyl-2-cyanoacrylate injection versus balloon-occluded retrograde transvenous obliteration. J Gastroenterol Hepatol.

[28] Hou MC, Lin HC, Lee HS, Liao WC, Lee FY, Lee SD (2009). A randomized trial of endoscopic cyanoacrylate injection for acute gastric variceal bleeding: 0.5 mL versus 1.0 mL. Gastrointest Endosc.

[29] Procaccini NJ, Al-Osaimi AM, Northup P, Argo C, Caldwell SH (2009). Endoscopic cyanoacrylate versus transjugular intrahepatic portosystemic shunt for gastric variceal bleeding: a single-center U.S. analysis. Gastrointest Endosc.

[30] Rivet C, Robles-Medranda C, Dumortier J, Le Gall C, Ponchon T, Lachaux A (2009). Endoscopic treatment of gastroesophageal varices in young infants with cyanoacrylate glue: a pilot study. Gastrointest Endosc.

[31] Cheng LF, Wang ZQ, Li CZ, Lin W, Yeo AE, Jin B (2010). Low incidence of complications from endoscopic gastric variceal obturation with butyl cyanoacrylate. Clin Gastroenterol Hepatol.

[32] Choudhuri G, Chetri K, Bhat G, Alexander G, Das K, Ghoshal UC (2010). Long-term efficacy and safety of N-butylcyanoacrylate in endoscopic treatment of gastric varices. Tropical Gastroenterol.

[33] Mishra SR, Chander Sharma B, Kumar A, Sarin SK (2010). Endoscopic cyanoacrylate injection versus beta-blocker for secondary prophylaxis of gastric variceal bleed: a randomised controlled trial. Gut..

[34] Soga K, Tomikashi K, Fukumoto K, Miyawaki K, Okuda K, Konishi H (2010). Successful endoscopic hemostasis for ruptured duodenal varices after balloon-occluded retrograde transvenous obliteration. Digestive Endoscopy.

[35] Binmoeller KF, Weilert F, Shah JN, Kim J (2011). EUS-guided transesophageal treatment of gastric fundal varices with combined coiling and cyanoacrylate glue injection (with videos). Gastrointest Endosc.

[36] Kang EJ, Jeong SW, Jang JY, Cho JY, Lee SH, Kim HG (2011). Long-term result of endoscopic Histoacryl (N-butyl-2-cyanoacrylate) injection for treatment of gastric varices. World J Gastroenterol.

[37] Liao SC, Yang SS, Ko CW, Lien HC, Tung CF, Peng YC (2013). A miniature ultrasound probe is useful in reducing rebleeding after endoscopic cyanoacrylate injection for hemorrhagic gastric varices. Scand J Gastroenterol.

[38] Tantau M, Crisan D, Popa D, Vesa S, Tantau A (2013). Band ligation vs. N-Butyl-2-cyanoacrylate injection in acute gastric variceal bleeding: a prospective follow-up study. Ann Hepatol.

[39] Al-Bawardy B, Gorospe EC, Saleem A, Buttar NS (2016). Wong Kee song LM. Outcomes and predictors of Rebleeding after 2-Octyl cyanoacrylate injection in acute gastric Variceal hemorrhage. J Clin Gastroenterol.

[40] Liu C, Ma L, Wang J, Li F, Tseng Y, Luo T (2019). Prophylactic use of antibiotics in endoscopic injection of tissue adhesive for the elective treatment of gastric varices: a randomized controlled study. J Gastroenterol Hepatol.

[41] Thakeb F, Salama Z, Salama H, Abdel Raouf T, Abdel Kader S, Abdel HH (1995). The value of combined use of N-butyl-2-cyanoacrylate and ethanolamine oleate in the management of bleeding esophagogastric varices. Endoscopy..

[42] Maruyama H, Okabe S, Ishihara T, Tsuyuguchi T, Yoshikawa M, Matsutani S (2010). Long-term effect of endoscopic injection therapy with combined cyanoacrylate and ethanol for gastric fundal varices in relation to portal hemodynamics. Abdom Imaging.

[43] Bhat YM, Weilert F, Fredrick RT, Kane SD, Shah JN, Hamerski CM (2016). EUS-guided treatment of gastric fundal varices with combined injection of coils and cyanoacrylate glue: a large U.S. experience over 6 years (with video). Gastrointest Endosc.

[44] Robles-Medranda C, Valero M, Nebel JA, de Britto Junior SR, Puga-Tejada M, Ospina J (2019). Endoscopic-ultrasound-guided coil and cyanoacrylate embolization for gastric varices and the roles of endoscopic Doppler and endosonographic varicealography in vascular targeting. Digestive Endoscopy.

[45] Zhang CQ, Liu FL, Liang B, Sun ZQ, Xu HW, Xu L (2008). A modified percutaneous transhepatic variceal embolization with 2-octyl cyanoacrylate versus endoscopic ligation in esophageal variceal bleeding management: randomized controlled trial. Dig Dis Sci.

[46] Tian X, Wang Q, Zhang C, Liu F, Cui Y, Liu F (2011). Modified percutaneous transhepatic variceal embolization with 2-octylcyanoacrylate for bleeding gastric varices: long-term follow-up outcomes. AJR Am J Roentgenol.

[47] Feretis C, Dimopoulos C, Benakis P, Kalliakmanis B, Apostolidis N (1995). N-butyl-2-cyanoacrylate (Histoacryl) plus sclerotherapy versus sclerotherapy alone in the treatment of bleeding esophageal varices: a randomized prospective study. Endoscopy..

[48] Dhiman RK, Chawla Y, Taneja S, Biswas R, Sharma TR, Dilawari JB (2002). Endoscopic sclerotherapy of gastric variceal bleeding with N-butyl-2-cyanoacrylate. J Clin Gastroenterol.

[49] Shi Y, Tian X, Hu J, Zhang J, Zhang C, Yang Y (2014). Efficacy of transjugular intrahepatic portosystemic shunt with adjunctive embolotherapy with cyanoacrylate for esophageal variceal bleeding. Dig Dis Sci.

[50] Ma LL, Luo TC, Tseng YJ, Huang XQ, Luo JJ, Zhang W (2018). Balloon-occluded retrograde Transvenous obliteration of Portovenous shunts during endoscopic therapy for the treatment of gastric Varices. Surg Laparoscopy Endoscopy Percutaneous Techniques.

[51] Dai YP, Gao Q (2017). A prognostic analysis of cirrhotic esophageal variceal bleeding treated with standardized endoscopic therapy. Zhonghua Gan Zang Bing Za Zhi.

[52] Zeng XQ, Ma LL, Tseng YJ, Chen J, Cui CX, Luo TC (2017). Endoscopic cyanoacrylate injection with or without lauromacrogol for gastric varices: a randomized pilot study. J Gastroenterol Hepatol.

[53] Sarin SK, Lahoti D, Saxena SP, Murthy NS, Makwana UK (1992). Prevalence, classification and natural history of gastric varices: a long-term follow-up study in 568 portal hypertension patients. Hepatology (Baltimore, Md).

